# Investigating the Impact of Aspartame on Ulcerative Colitis Through Network Toxicology and Molecular Docking

**DOI:** 10.1002/fsn3.70869

**Published:** 2025-08-28

**Authors:** Lili Ge, Lei Ma, Zhi Jin

**Affiliations:** ^1^ Department of Traditional Chinese Medicine The Second Hospital of Shandong University Jinan China; ^2^ Department of Pharmacy Department Shanghe County People's Hospital Jinan China

**Keywords:** aspartame, network toxicology, ulcerative colitis

## Abstract

The aim of this study is to elucidate the impact of aspartame on ulcerative colitis (UC). The research methodology involved extracting aspartame‐associated targets from the ChEMBL and STITCH databases, while potential UC‐related targets were obtained from the OMIM and GeneCards databases. The STRING platform and Cytoscape software were utilized to identify key targets through which aspartame may exert its influence on UC. Comprehensive analyses of Gene Ontology (GO) and Kyoto Encyclopedia of Genes and Genomes (KEGG) pathways were conducted using the DAVID database. Furthermore, molecular docking simulations were employed to validate the binding interactions between aspartame and these key targets. A total of 297 targets associated with aspartame and 5968 targets related to UC were identified. Protein–protein interaction (PPI) analysis indicated that BCL2, ESR1, HSP90AA1, SRC, TNF, and CASP3 may serve as central targets through which aspartame affects UC. KEGG pathway analysis highlighted the involvement of pathways such as the C‐type lectin receptor, sphingolipid, and VEGF signaling pathways. In molecular docking studies, a binding energy of less than −5.0 kcal mol^−1^ is indicative of strong binding activity. The results of the molecular docking analysis demonstrated that aspartame exhibits a high binding affinity with proteins including BCL2, ESR1, HSP90AA1, SRC, TNF, and CASP3. These interactions suggest that aspartame may play a role in the initiation and progression of UC through multiple targets and signaling pathways. Nonetheless, given the complexity of in vivo environments, it is crucial to corroborate these molecular findings within biological systems. This study offers a theoretical foundation that now needs to be confirmed through experimental and clinical research.

## Graphical abstract

Through the application of network toxicology and molecular ligation, this study elucidates that aspartame may influence the progression of ulcerative colitis by targeting molecules such as BCL2, TNF, and CASP3, thereby modulating pathways related to inflammation and apoptosis, including the C‐type lectin receptor, sphingolipids, and VEGF pathways. These findings offer valuable insights into the potential health implications of aspartame and lay the groundwork for future research endeavors.

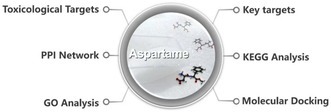

## 1 Introduction

Ulcerative colitis (UC) is a chronic inflammatory condition that primarily affects the rectum and colon (Ungaro et al. 2017). As of 2023, the estimated global prevalence of UC is approximately 5 million cases, with a rising incidence observed worldwide (Le Berre et al. 2023). The etiology of UC remains unclear; however, it is believed to be influenced by a combination of genetic predisposition, environmental factors, luminal conditions, and dysregulation of the mucosal immune system (Kobayashi et al. 2020; Zangara et al. 2022). Recent research has identified aspartame as promoting intestinal pathology and systemic inflammation (Keshteli et al. 2019; Laudisi et al. 2019; Levine et al. 2020; Zhong et al. 2024).

Aspartame, a widely used artificial sweetener found in low‐calorie products, is generally regarded as safe. However, emerging research suggests that it may exacerbate inflammatory responses in UC by disrupting intestinal immune function, increasing oxidative stress, and compromising the integrity of the intestinal barrier (Shil et al. 2020; Zhong et al. 2024). Although there is no consensus on this issue, accumulating evidence indicates that aspartame may induce oxidative stress and damage cellular membranes, potentially leading to cellular dysfunction and systemic inflammation (Choudhary and Pretorius 2017). Given the widespread consumption of aspartame in food products and its long‐term use, it is crucial to investigate its effects on the development of UC, particularly concerning immune regulation and gut microbiota, which are of significant scientific and clinical importance (Levine et al. 2020).

This study aims to investigate the potential effects of aspartame on UC by examining mechanisms such as alterations in gut microbiota, immune responses, and intestinal barrier function. Understanding these mechanisms is essential for developing effective strategies to mitigate the potential adverse health effects of aspartame and to enhance clinical management for patients with UC. The primary objective of this research is to assess the possible influence of aspartame on the pathogenesis of UC through various analytical methods. To achieve this, we integrated multiple data sources to identify differentially expressed genes and immune markers associated with aspartame exposure. Additionally, we conducted enrichment analyses to identify key biological pathways and mechanisms related to the effects of aspartame. Finally, we explored the interactions between aspartame metabolites and critical immune‐related proteins using molecular docking techniques. This research elucidates how aspartame may contribute to the development of UC through molecular mechanisms and establishes a theoretical foundation for potential future clinical interventions. Our findings underscore the critical need to evaluate food additives in the context of chronic intestinal diseases, thereby contributing to the development of more informed and precise public health policies.

## 2 Methods

### 2.1 Identification of Toxicological Targets for Aspartame

Utilize the online data platform PubChem (https://pubchem.ncbi.nlm.nih.gov/) to acquire the SMILES notation for the compound of interest. Subsequently, import this information into the ChEMBL database (https://www.ebi.ac.uk/chembl/) and the STITCH database (http://stitch.embl.de/), ensuring the species is set to *Homo sapiens* and the minimum required interaction score is configured to 0.4, to identify human targets associated with aspartame.

### 2.2 Identification of Targets for UC

Utilizing “Ulcerative colitis (UC)” as the search term, disease‐associated targets were compiled from the OMIM database (https://omim.org/) and the GeneCards database (https://www.genecards.org/), with the latter requiring a relevance score of 0.25 or higher. The BMK Cloud platform was utilized to identify overlapping targets between aspartame and UC, resulting in a Venn diagram that illustrates the intersecting targets.

### 2.3 Construction of the Protein–Protein Interaction Network

The STRING database was used to analyze the protein interaction network of the overlapping targets. In this study, the target associated with aspartame was aligned with the target relevant to ulcerative colitis. The results of this mapping were subsequently imported into the STRING database, with multiple proteins and *Homo sapiens* specified as parameters. To enhance the representation of the relationships between the targets, the minimum interaction score was established at 0.4, facilitating the construction of the protein interaction network. Key targets were identified using the “CytoHubba” plugin in Cytoscape, which employs five distinct algorithms: Betweenness Centrality, Closeness Centrality, Degree Centrality, Maximum Clique Centrality (MCC), and Edge Percolated Component (EPC).

### 2.4 GO And KEGG Enrichment Analyses

Aspartame's interaction with ulcerative colitis was analyzed using mutual mapping results imported via the online software DAVID (https://david.ncifcrf.gov/). The analysis was conducted with the parameters set to *Homo sapiens* and a significance threshold of *p* < 0.05. GO and KEGG analyses were performed on the obtained data by selecting GO Biological Processes, GO Molecular Functions, GO Cellular Components, and KEGG pathways. The enrichment results from the GO and KEGG pathway analyses were visualized based on *p*‐values to facilitate interpretation.

### 2.5 Molecular Docking

**FIGURE 1:**
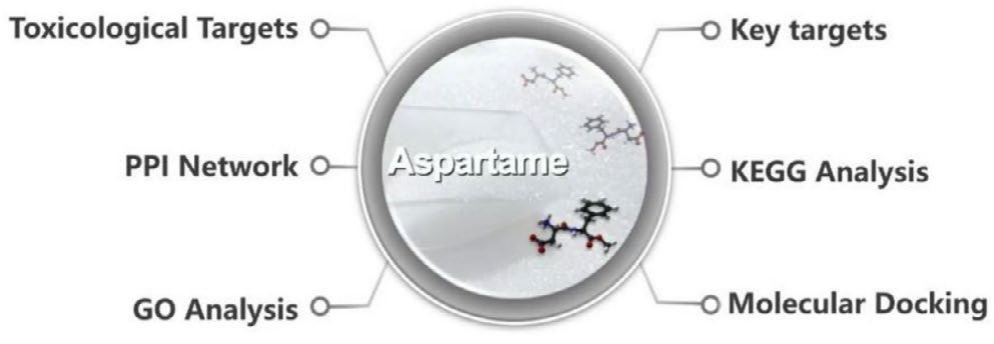
Framework diagram.

The two‐dimensional structures of small molecular ligands were obtained from the PubChem database (http://pubchem.ncbi.nlm.nih.gov/). These structures were subsequently converted into three‐dimensional formats using ChemOffice and saved as .mol2 files. The high‐resolution crystal structure of the protein target was retrieved from the RCSB Protein Data Bank (http://www.rcsb.org/) to serve as the molecular docking receptor. Prior to docking, several pretreatment steps, including protein dehydration and dephosphorylation, were performed using PyMOL software, and the processed structures were saved as PDB files. Molecular docking was conducted using AutoDock Vina version 1.5.6 to perform semi‐flexible targeted docking, facilitating the study of interactions between the proteins and ligands. The exhaustiveness value was set to 8, which is the default setting of the software, and 10 output conformations were generated for each ligand. The mesh parameters were configured based on the coordinates of the co‐crystalline ligand's center. AutoDock facilitated the preparation of protein and small molecule structures, incorporating processes such as protein hydrogenation and dehydration, hydrogenation of small molecule ligands, and the assessment of torsional degrees of freedom. To establish the coordinates of the docking box, first customize the grid size according to the spatial characteristics of the active pocket. Subsequently, execute the docking process and save all conformations to a PDBQT file. For ESR, the exhaustiveness value is set to 8, with a grid box centered at (61.443, 43.105, 68.8) and dimensions of 46.5 × 47.25 × 47.25 Å, covering active sites from co‐crystallized ligands or literature. Other targets use similar methods with an exhaustiveness value of 8. HSP90AA1's grid is centered at (34.004, −48.832, 61.519) with dimensions of 47.25 × 42.75 × 47.25 Å. CASP3's grid is centered at (14.293, 1.286, 33.285) with dimensions of 47.25 × 47.25 × 47.25 Å. BCL2's grid is centered at (47.359, 33.217, −5.437) with dimensions of 43.5 × 47.25 × 47.25 Å. TNF's grid is centered at (0.178, 32.124, 53.949) with dimensions of 47.25 × 47.25 × 47.25 Å. SRC's grid is centered at (44.788, 18.065, 19.232) with dimensions of 47.25 × 47.25 × 38.25 Å. The analysis of results identified the conformation with the lowest binding energy (ΔG, kcal/mol) as the optimal outcome (Figure 1). Two‐dimensional and three‐dimensional visualization analyses of the interactions between the test compounds and the key residues were conducted utilizing PyMOL and Discovery Studio 2019 software. All target sites were determined based on literature reports or co‐crystalline ligand sites.

## 3 Results

### 3.1 Identification of Aspartame‐Induced Targets in UC

The chemical structure of aspartame, along with its SMILES code, was obtained from the PubChem database. By integrating human targets associated with aspartame from the ChEMBL and STITCH databases, a total of 297 targets were identified. Concurrently, gene information relevant to UC was consolidated from the GeneCards and OMIM databases, resulting in 5968 disease‐related gene targets after the removal of duplicates. Utilizing an online bioinformatics mapping platform, 147 intersecting target genes between aspartame and UC were identified. These intersecting targets may serve as potential candidates for aspartame‐induced UC.

### 3.2 Interaction Network of Potential Targets and Core Gene Acquisition

**FIGURE 2:**
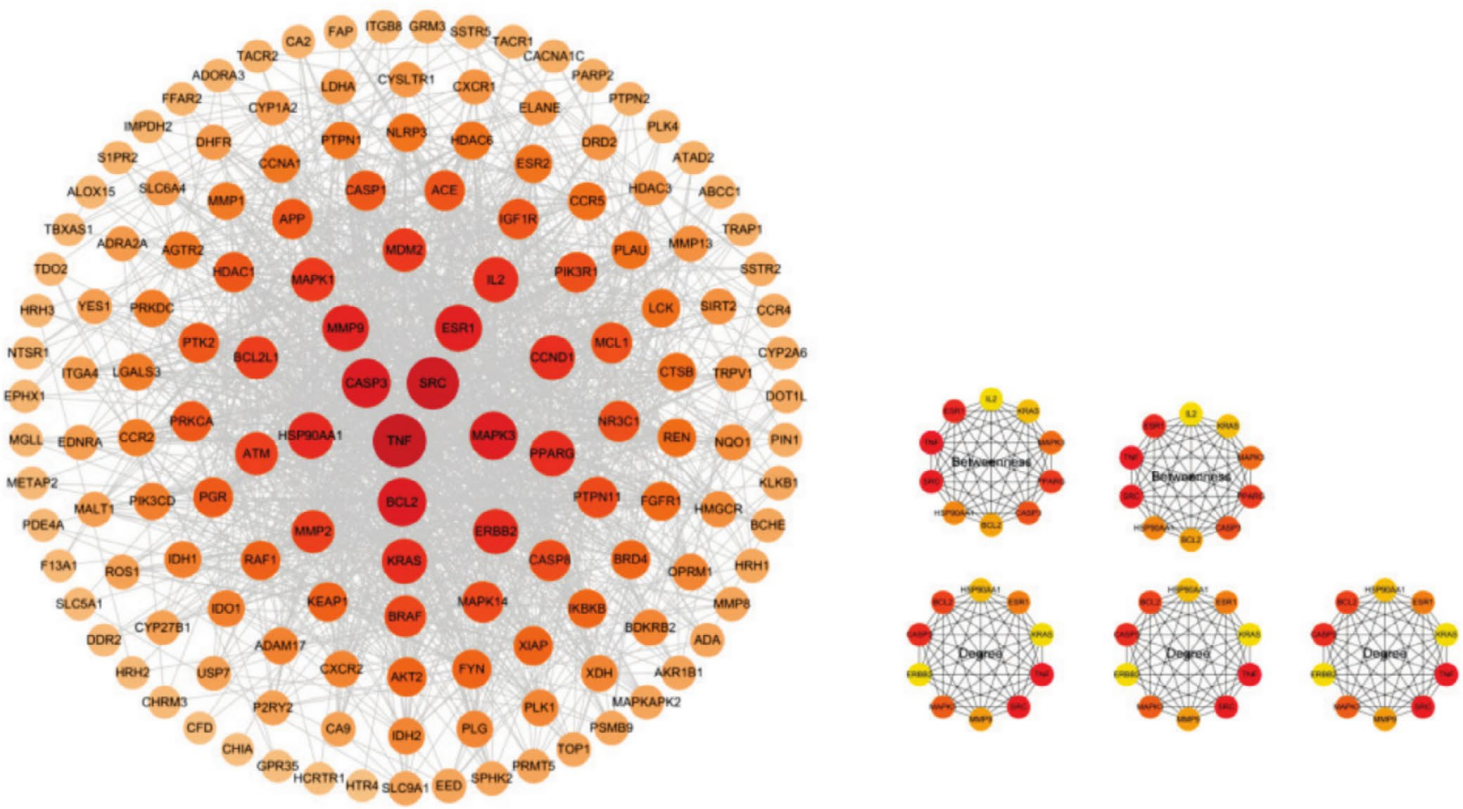
The PPI network of the potential targets and the core targets.

**FIGURE 3:**
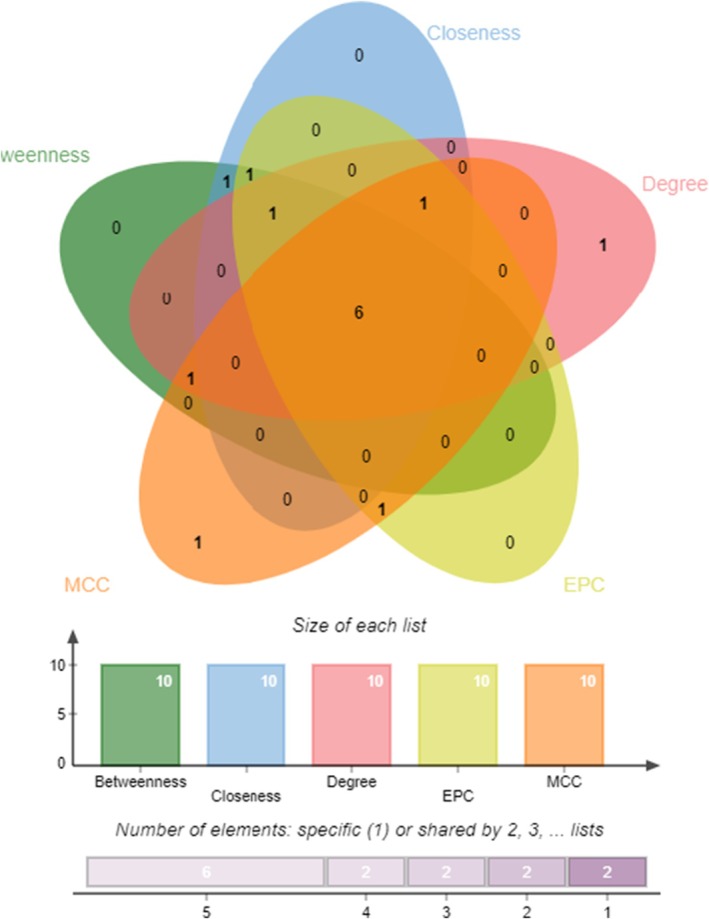
Venn diagram of the core target.

The 147 intersecting targets were analyzed using the STRING database, which revealed 1483 edges and 147 nodes (Figure 2). A protein–protein interaction (PPI) network was subsequently constructed using Cytoscape software. Further analysis with the CytoHubba plugin, employing five distinct algorithms, identified six core targets: BCL2, ESR1, HSP90AA1, SRC, TNF, and CASP3. These targets are pivotal within the PPI network and are essential for understanding aspartame‐induced UC (Figure 3).

### 3.3 Target and Pathway Enrichment Analysis

**FIGURE 4:**
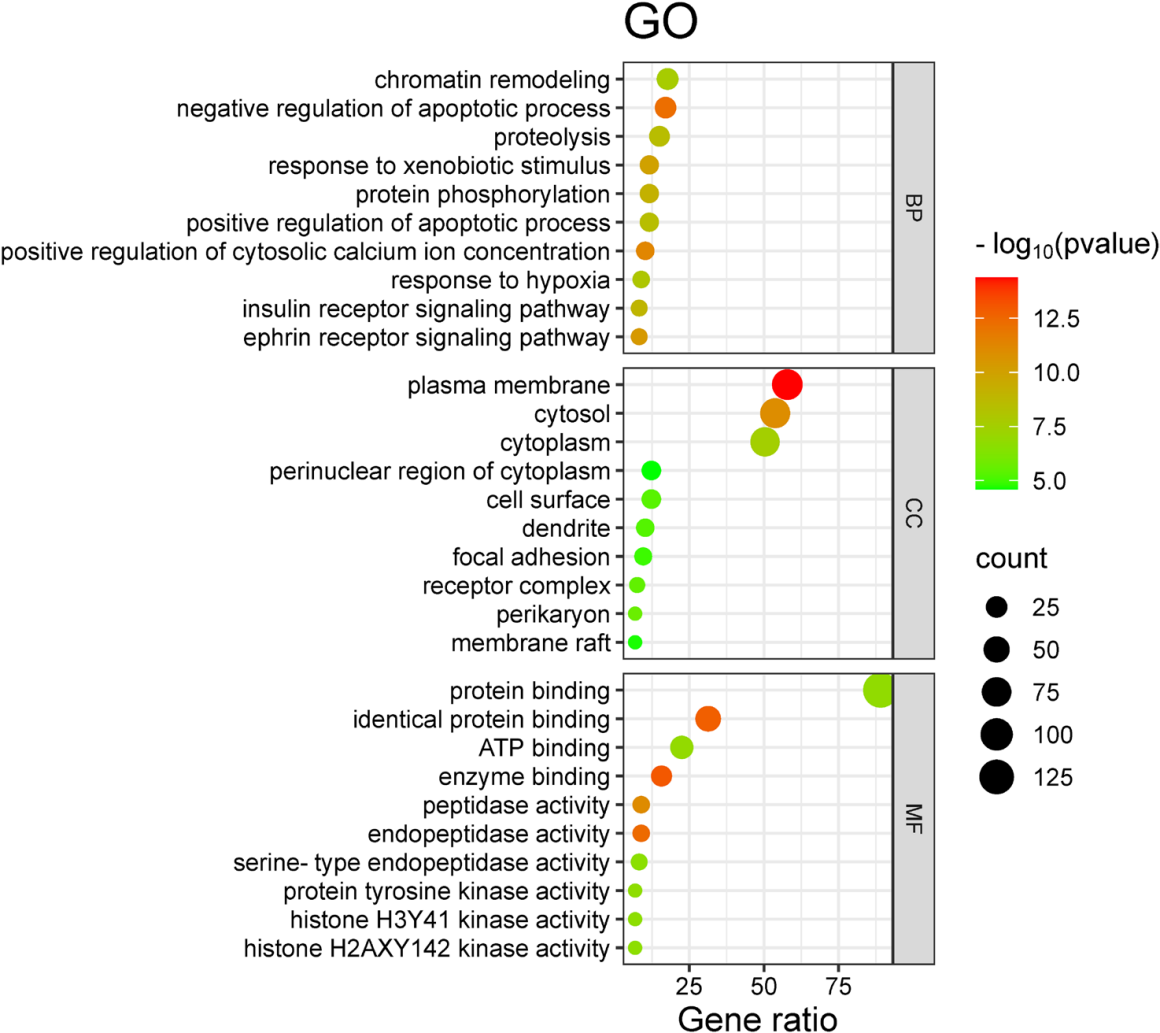
GO enrichment analysis of potential targets.

**FIGURE 5:**
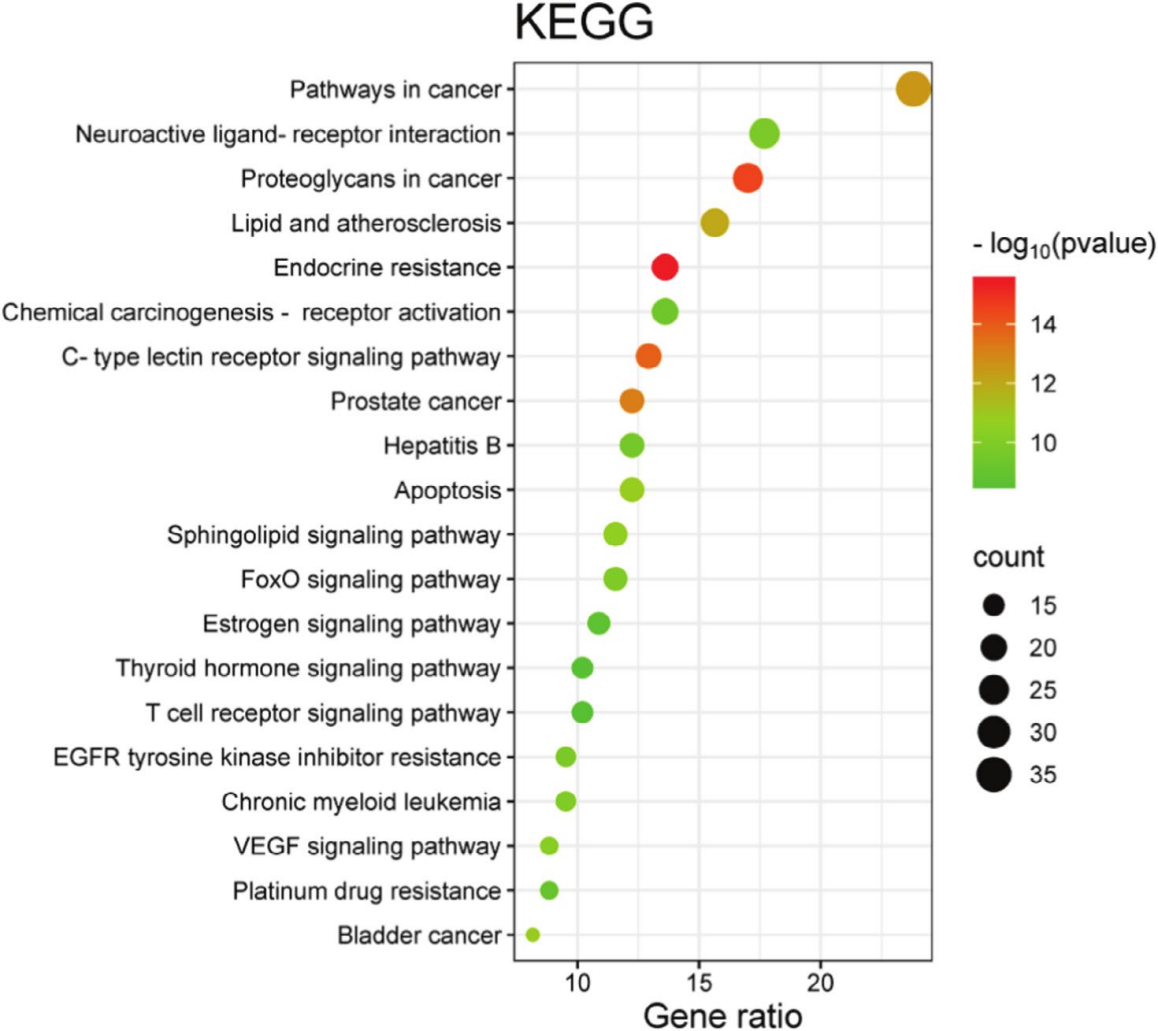
KEGG enrichment analysis of potential targets.

**FIGURE 6:**
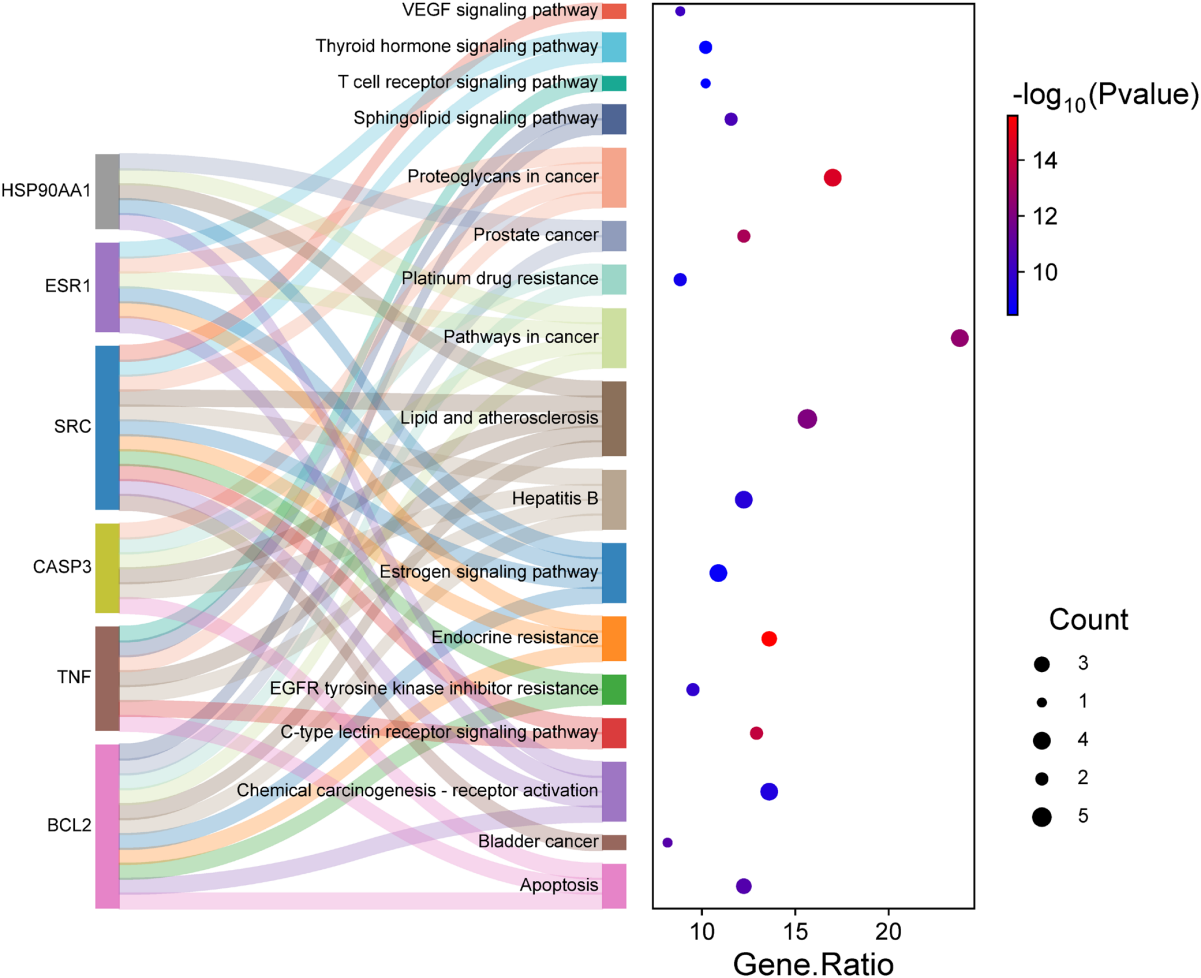
Mulberry map of enrichment of core targets.

To investigate the role of aspartame in the onset and progression of UC, GO enrichment analysis was conducted on 147 intersecting target genes using the DAVID online database. This analysis identified a total of 749 statistically significant GO terms (*p* < 0.05), which included 353 Biological Process (BP) terms, 54 Cellular Component (CC) terms, and 140 Molecular Function (MF) terms. The findings suggest that the target genes are primarily involved in biological processes such as apoptosis, anti‐inflammatory responses, and antioxidant activities. The CC analysis indicated that these genes predominantly function at the plasma membrane, cytoplasm, and cytosol. The MF analysis revealed that the targets mainly participate in binding to enzymes, proteins, and protein kinases. Furthermore, KEGG pathway analysis demonstrated that the target genes were enriched in 146 related pathways, primarily involving C‐type lectin receptors, sphingolipids, and vascular endothelial growth factor (VEGF), which are closely associated with apoptosis, oxidative stress, and inflammatory responses (Figures 4, 5, 6).

### 3.4 Molecular Docking

**FIGURE 7:**
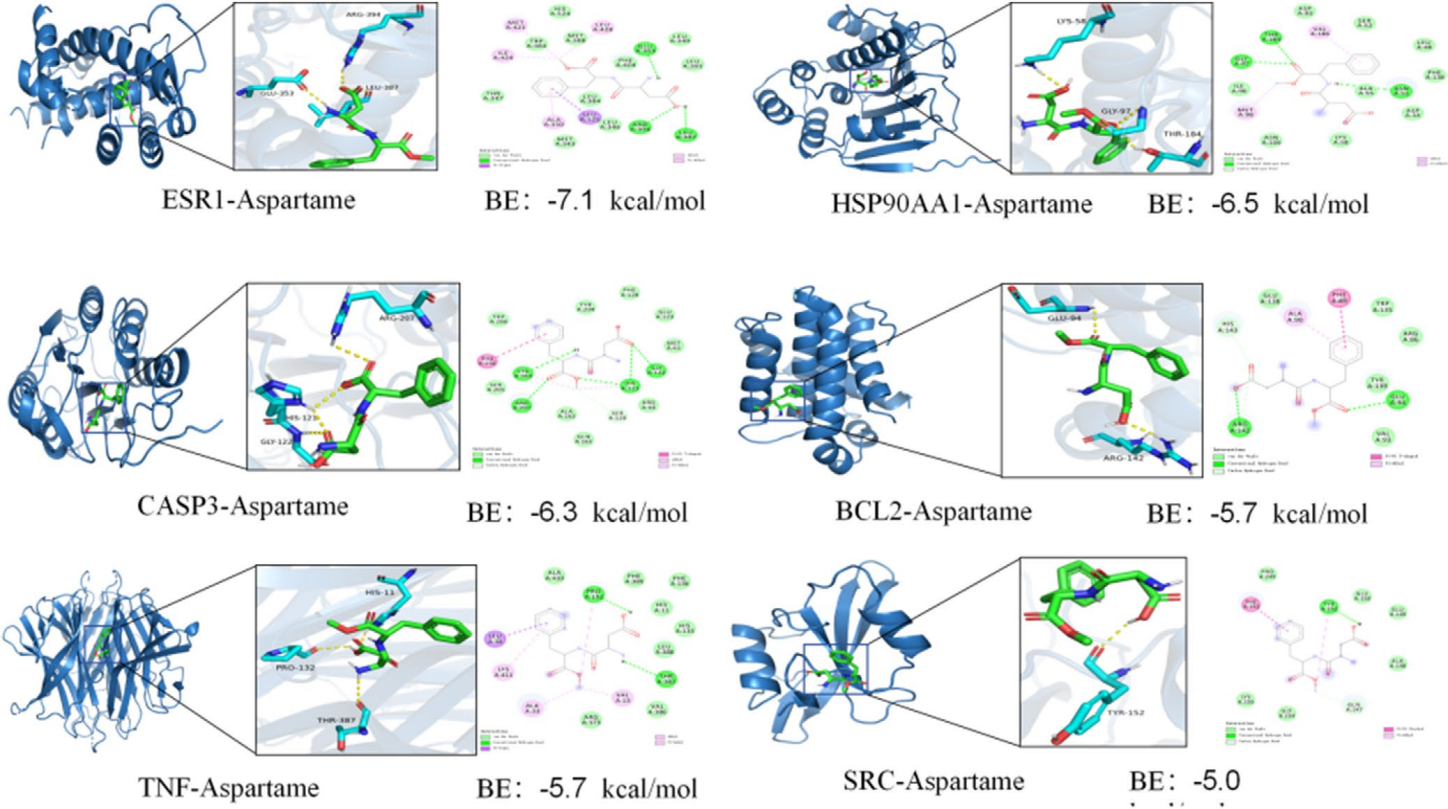
The results of molecular docking.

Molecular docking simulations were conducted to evaluate the interaction between aspartame and the core target proteins, utilizing AutoDock software to predict binding affinities. A lower binding energy indicates a more stable molecular conformation; generally, a binding energy of less than 0 kcal·mol^−1^ suggests molecular binding activity, while a binding energy below −5.0 kcal·mol^−1^ denotes good binding activity. Upon docking aspartame with the six target proteins, all exhibited favorable binding potential. In the ESR1 receptor, the ARG394, LEU387, and GLU353 residues establish hydrogen bond interactions with aspartame, while the ALA350, LEU428, MET421, and ILE424 residues engage in hydrophobic interactions with the compound. Similarly, the HSP90AA1 receptor's GLY97, THR184, and ASN51 residues form hydrogen bond interactions with aspartame, whereas the MET98 and VAL186 residues participate in hydrophobic interactions. In the CASP3 receptor, the ARG207, CYS163, HIS121, and GLY122 residues are involved in hydrogen bonding with aspartame, and the CYS162 and HIS121 residues contribute to hydrophobic interactions. The BCL2 receptor features ARG142 and GLU94 residues that form hydrogen bonds with aspartame, and the ALA90 residue engages in a hydrophobic interaction. The TNF receptor's PRO132 and THR387 residues form hydrogen bonds with aspartame, while LYS411, ALA33, and VAL13 residues participate in hydrophobic interactions. Lastly, the TYR152 residue on the SRC receptor forms a hydrophobic interaction with aspartame. Visualization of the docking conformations was performed using PyMOL software, revealing that aspartame interacts with each core target protein, thereby playing a potential role in the molecular mechanism underlying induced UC (Figure 7 and Table 1).

**TABLE 1:** Binding capacity of aspartame to core targets (Kcal·mol^−1^).

NO.	Ingredients	Target	Binding Affinity (kcal/mol)
1	Aspartame	ESR1	−7.1
2	Aspartame	HSP90AA1	−6.5
3	Aspartame	CASP3	−6.3
4	Aspartame	BCL2	−5.7
5	Aspartame	TNF	−5.7
6	Aspartame	SRC	−5

## 4 Discussion

UC is a chronic inflammatory bowel disease that primarily affects the colon and rectum (Hirten and Sands 2021). It is characterized by symptoms such as abdominal pain, diarrhea, weight loss, and rectal bleeding (Kobayashi et al. 2020). The exact etiology of this condition remains incompletely understood; however, it is believed to involve a combination of genetic predispositions, immune system dysfunctions, and environmental factors. Aspartame, a widely used artificial sweetener found in low‐calorie and sugar‐free products, has been the subject of ongoing debate regarding its safety and potential health effects. Dietary and lifestyle modifications are essential for managing UC, and research has suggested that artificial sweeteners, including aspartame, may negatively impact gut health, particularly in individuals with pre‐existing intestinal disorders (Choudhary and Pretorius 2017; Keshteli et al. 2019; Shil et al. 2020; Zhong et al. 2024). Consequently, exploring the relationship between aspartame consumption and UC has become a significant focus in the fields of nutrition and medical research.

This study conducted a multi‐database integrative analysis to elucidate the potential mechanisms by which aspartame influences UC. The analysis utilized databases such as ChEMBL and STITCH, resulting in the identification of 297 targets associated with aspartame. Additionally, 5968 potential targets related to UC were extracted from the OMIM and GeneCards databases. Subsequent analysis of these targets using the STRING platform and Cytoscape software revealed six core targets (BCL2, ESR1, HSP90AA1, SRC, TNF, and CASP3) as significant contributors to aspartame‐induced UC.

UC is characterized by a persistent inflammatory process that depends on cytokine production, with TNF serving as a pivotal regulatory element in the inflammatory response (Jałocha et al. 2009). TNF‐α is significantly overexpressed in the intestinal mucosa of patients with UC, where it activates nuclear factor kappa B (NF‐κB) to induce the expression of pro‐inflammatory genes and stimulate the caspase cascade, thereby promoting apoptosis (Yang et al. 2013). Research has demonstrated that anti‐TNF‐α agents are essential therapeutic options for UC, showing both efficacy and safety in extensive randomized controlled trials. The mechanism of action of these agents may involve the inhibition of apoptosis in intestinal epithelial cells (IECs) (Samaan et al. 2014). Studies have indicated that aspartame can increase levels of the pro‐inflammatory factor TNF‐α in intestinal tissues and serum, thereby exacerbating the inflammatory microenvironment of the intestine (Choudhary and Pretorius 2017). The balance of apoptosis is jointly regulated by B‐cell lymphoma 2 (BCL2) and caspase 3 (CASP3), and the disruption of the intestinal epithelial barrier in UC is closely associated with excessive apoptosis (Lebda et al. 2017). BCL2 and CASP3 collaboratively modulate the equilibrium of apoptosis, with excessive apoptosis being closely associated with the disruption of the intestinal epithelial barrier in UC. The inhibition of BCL2 coupled with the activation of CASP3 may result in the uncontrolled apoptosis of intestinal epithelial cells, thereby undermining mucosal barrier integrity. Studies have demonstrated that aspartame can induce the upregulation of Bax, Casp3, P27, and MDM2 expression while concurrently downregulating BCL2 expression, indicating the activation of apoptotic pathways (Lebda et al. 2017). ESR1, a nuclear hormone receptor, exhibits a reciprocal inhibitory relationship with NF‐κB and may also contribute to the activation of NOS3 and the production of endothelial nitric oxide (Kim et al. 2011). The dysregulation of estrogen receptors within the intestinal mucosa in UC suggests that estrogen signaling may modulate local immune responses (Jacenik et al. 2019; Wu et al. 2022; L. Xu et al. 2022). Src exhibits potential anti‐inflammatory properties in the context of inflammatory diseases, as its activation can inhibit NF‐κB and enhance activator protein 1 (AP‐1) activation, thereby leading to a reduction in TNF‐α levels (Hu et al. 2016). Empirical evidence has demonstrated that the Src inhibitor dasatinib exacerbates colitis by elevating TNF‐α levels, which occurs through the suppression of IL‐10, possibly involving the activation of the Src‐Akt signaling pathway to facilitate IL‐10 production (Hu et al. 2016). HSP90AA1, functioning as a molecular chaperone, modulates the stability of various signaling proteins, including AKT and NF‐κB, which may contribute to apoptosis in colorectal cancer cells by regulating inflammation and stress responses (Liu et al. 2025; Y. Xu et al. 2025; Zhang, Huang, et al. 2022; Zhang, Peng, et al. 2022). Molecular docking studies have revealed that these six core target proteins exhibit effective binding with aspartame, suggesting the hypothesis that aspartame may influence the onset and progression of UC through interactions with these core proteins. This study employed molecular docking to predict the binding mode of aspartame with potential targets, providing computational support for understanding its biological activity. However, the practical significance of these findings should be approached with caution. Molecular docking, as a computational simulation tool, is primarily valuable for generating testable hypotheses rather than confirming binding interactions. Its limitations include reliance on the static conformation of protein crystal structures, which fails to capture the dynamic conformational changes occurring under physiological conditions. Additionally, the simplification of solvation models and the estimation of entropy changes can lead to inaccuracies in binding energy calculations. Furthermore, molecular docking lacks a biological context and does not account for critical factors influencing actual binding, such as cell membrane permeability, in vivo concentration, and metabolic transformation. Consequently, the current docking results serve as preliminary evidence suggesting that aspartame may interact with the target. These predictions require experimental validation to confirm their biological relevance (Uba and Zengin 2023).

It is important to highlight that while this study has identified targets associated with ulcerative colitis (UC), such as TNF, ESR1, and HSP90AA1, the fundamental pathology of UC is driven by more specific factors and biomarkers. For example, the decreased expression of mucin MUC2, a crucial component of the colonic mucus layer, compromises barrier function and serves as a significant driver in the development of UC. Additionally, Interleukin‐23 (IL‐23) plays a pivotal role as a regulatory factor in autoimmune inflammation; its excessive activation directly induces intestinal inflammation, and therapies targeting IL‐23 have demonstrated substantial efficacy. However, bioinformatics analyses have inherent limitations in elucidating protein functions and causal relationships, which restrict the comprehensive exploration of core mechanisms such as MUC2 barrier integrity and IL‐23 pathway activity. Consequently, future research should focus on directly validating these core targets and their associations with the targets identified in this study through clinical sample testing and functional experiments, to more thoroughly elucidate the mechanisms underlying UC.

Subsequently, an enrichment analysis of the target proteins was conducted to investigate the primary signaling pathways and specific mechanisms through which aspartame influences UC. The results of this analysis revealed that the target proteins are involved in multiple signaling pathways, predominantly including C‐type lectin receptors, sphingolipid signaling, and vascular endothelial growth factor (VEGF) signaling pathways. C‐type lectin‐like receptors are glycan‐binding receptors that can modulate the activity of intraepithelial lymphocytes, thereby influencing the reactivity and integrity of the epithelial barrier (Leibelt et al. 2015). Tumor necrosis factor (TNF) can upregulate the DECTIN2 family of C‐type lectin receptors in human myeloid cells, and impaired expression of these receptors in patients undergoing TNF‐blocking therapy may hinder microbial perception and defense against infections (Haberkamp et al. 2023). Complex sphingolipids are essential structural components of the intestinal membrane, playing a crucial role in safeguarding the intestinal mucosal barrier and regulating the intestinal absorption process (Abdel Hadi et al. 2016). Numerous studies have affirmed the pivotal role of intestinal sphingolipids in immune and inflammatory diseases through their involvement in signaling pathways that affect the survival, growth, differentiation, and apoptosis of intestinal cells (Abdel Hadi et al. 2016; Espinoza and Snider 2024). Sphingolipid signaling is acknowledged as a significant pro‐inflammatory mediator that regulates the tumor necrosis factor‐alpha (TNF‐α) signaling pathway (Sukocheva et al. 2020). The anti‐inflammatory cytokine interleukin‐10 (IL‐10) modulates sphingolipid metabolism to attenuate nuclear factor kappa‐light‐chain‐enhancer of activated B cells (NF‐κB)‐mediated inflammation, thereby increasing the risk of inflammatory bowel disease (Ryan and Zanoni 2024). Conversely, restoring normal sphingolipid metabolism can mitigate this risk. The sphingolipid network exhibits high sensitivity to various specific metabolic alterations, including those involving amino acids (such as serine and alanine), fatty acids, choline (and ethanolamine), and glucose (Ryan and Zanoni 2024). Research on angiogenesis and lymphangiogenesis holds significant implications for the pathogenesis of UC, with chronic intestinal inflammation being associated with pathological changes (Linares and Gisbert 2011). Angiogenesis plays a critical role in exacerbating the inflammatory response, with vascular endothelial growth factor (VEGF) identified as a key angiogenic cytokine associated with chronic colitis and inflammatory bowel disease (Ardelean et al. 2014; Kopanakis et al. 2014). Research conducted by Zhong et al. has demonstrated that aspartame elevates the levels of pro‐inflammatory cytokines, including TNF‐α, IL‐1*β*, and IL‐6, in both intestinal tissues and serum. Furthermore, aspartame has been shown to increase the infiltration of macrophages and neutrophils into colonic tissues. These findings suggest that aspartame may contribute to pathological changes in the intestine and systemic inflammation in a murine model of colitis (Choudhary and Pretorius 2017). Additionally, Shalaby et al. have indicated that aspartame may modulate the expression of vascular endothelial growth factor under various conditions, thereby promoting angiogenesis and influencing the onset of disease (Yesildal et al. 2015). Moreover, research by Shil et al. has confirmed that aspartame affects the permeability of intestinal epithelial cells, with high concentrations leading to apoptosis and cell death in these cells (Shil et al. 2020).

This study investigated the potential molecular mechanisms and biological effects of aspartame utilizing bioinformatics approaches. It is important to emphasize that current computational analyses, including molecular docking and pathway enrichment, primarily provide predictions at the molecular or pathway level and do not account for actual dose–response relationships. Furthermore, no exposure concentrations corresponding to actual human dietary intake or the acceptable daily intake (ADI) were established. Consequently, the findings of this study, such as predicted binding affinities and affected signaling pathways, cannot be directly extrapolated or quantitatively assessed for their biological significance or health risks in the context of daily human aspartame consumption, particularly within the ADI range. Future research should integrate in vitro experiments employing physiologically relevant concentrations and population‐based epidemiological studies assessing various exposure levels to validate the molecular effects and potential impacts of these predictions in scenarios more representative of real‐world exposure. Although our analysis indicates potential associations between aspartame and various inflammation‐related targets and signaling pathways, these findings necessitate validation through clinical studies. While these computational insights are promising, their biological significance remains hypothetical until supported by experimental validation. A comprehensive assessment of the long‐term effects of aspartame on UC can only be achieved through real‐world data and rigorous experimental validation.

## 5 Conclusion

This study suggests that aspartame may influence the onset and progression of UC by modulating inflammatory responses, promoting apoptosis and angiogenesis, and compromising barrier protection through various related targets and pathways. This conclusion is supported by the integration and analysis of multiple databases. Although these initial insights into the molecular mechanisms provide a foundation for further exploration, additional validation through experimental studies and clinical data is essential. This study suggests a possible role of aspartame in UC pathogenesis, which should be further explored through preclinical and clinical studies.

## Author Contributions

**Lili Ge:** conceptualization (equal), formal analysis (equal), writing – original draft (equal). **Lei Ma:** data curation (equal), investigation (equal), methodology (equal), software (equal), validation (equal), visualization (equal). **Zhi Jin:** conceptualization (equal), data curation (equal), project administration (equal), resources (equal), software (equal), supervision (equal), writing – review and editing (equal).

## Conflicts of Interest

The authors declare no conflicts of interest.

## Data Availability

Data Availability StatementThe data that support the findings of this study are available from the corresponding authors upon reasonable request.
